# T Cells and Acute Kidney Injury: A Two-Way Relationship

**DOI:** 10.3389/fimmu.2020.01546

**Published:** 2020-07-17

**Authors:** Sergio Dellepiane, Jeremy S. Leventhal, Paolo Cravedi

**Affiliations:** ^1^Icahn School of Medicine at Mount Sinai, New York, NY, United States; ^2^Division of Nephrology, White Plains Hospital, White Plains, NY, United States

**Keywords:** AKI, IRI, regulatory T cell, Treg, TH1, TH2, TH17

## Abstract

Acute Kidney Injury (AKI) complicates up to 10% of hospital admissions substantially increasing patient morbidity and mortality. Experimental evidence supports that AKI initiation and maintenance results from immune-mediated damage. Exogenous injury sources directly damage renal cells which produce pro-inflammatory mediators recruiting immune cells and furthering kidney injury. Many AKI studies focus on activation of innate immunity; major components include complement pathways, neutrophils, and monocytes. Recently, growing evidence emphasizes T lymphocytes role in affecting AKI pathogenesis and magnitude. In particular, T helper 17 lymphocytes enhance tissue injury by recruiting neutrophils and other inflammatory cells, while regulatory T cells conversely reduce renal injury and facilitate repair. Intriguingly, evidence supports local parenchymal-T cell interactions as essential to producing T cell phenotypic changes affecting long-term kidney and patient survival. Herein, we review T cells effects on AKI and patient outcomes and discuss related new therapeutic approaches to improve outcomes of affected individuals.

Acute kidney injury (AKI) is clinically defined by rapid renal function decline indicated by serum creatine rise ≥0.3 mg/dl (or >50% from baseline) and/or urine output ≤500 ml/day (1). It is classified as pre-renal, post-renal or parenchymal (also known as intrinsic) depending on the primary site of injury. Pre-renal and post-renal AKI are consequences of altered renal perfusion or urinary tract obstruction, respectively; thus, they represent extrinsic disorders. However, if pre/post-renal injuries persist, AKI will eventually evolve to cellular damage and intrinsic kidney disease. Pathophysiologically, AKI represents complex interactions of exogenous injury and host responses culminating in decreased glomerular filtration.

In the last decade, new approaches focused on more specific nomenclature across types of parenchymal AKI (2). Indeed, while pre- and post-renal AKI are frequently reversible and minimally impactful on patient survival (3), parenchymal AKI is an emerging global health concern, increases patient morbidity/mortality risk, and rose in incidence over the last 30 years (4). In industrialized countries, AKI affects 5–10% of hospitalized patients and 25–50% of those in intensive care units (ICU) (4, 5). A 2013 meta-analysis estimated that mortality rates for hospital-acquired AKI is ~23% and rises to 50% in subsets requiring dialysis (5). Similarly, a large registry study on >190,000 patients reported 90-day AKI mortality rates of 37% (vs. 7% in non-AKI group). In the same cohort 2 years post-discharge, AKI survivors' combined risk of death, end stage renal disease (ESRD) or chronic kidney disease (CKD) was >30%, more than double of the cohort without AKI (6, 7).

Taken together, clinical data and experimental animal AKI models, concur that AKI associates or contributes to lung, heart, liver, brain, or gut damage (8) that produces long-term sequelae in affected organs (9). Importantly, immune system function is tightly linked to AKI with bidirectional influence. While sepsis is a recognized leading cause of hospital-associated kidney injury (4), AKI also associates with increased infection risk even after full recovery of renal function (10, 11). The first studies about immune cell activation during AKI focus on innate immune response; more recently research shows adaptive immunity activation during AKI contributing to renal and extra-renal outcomes. Herein, we will review both adaptive immune contributions to AKI and immune function changes related to AKI.

## Etiologies of Hospital Related Acute Kidney Injury

**Figure 1:**
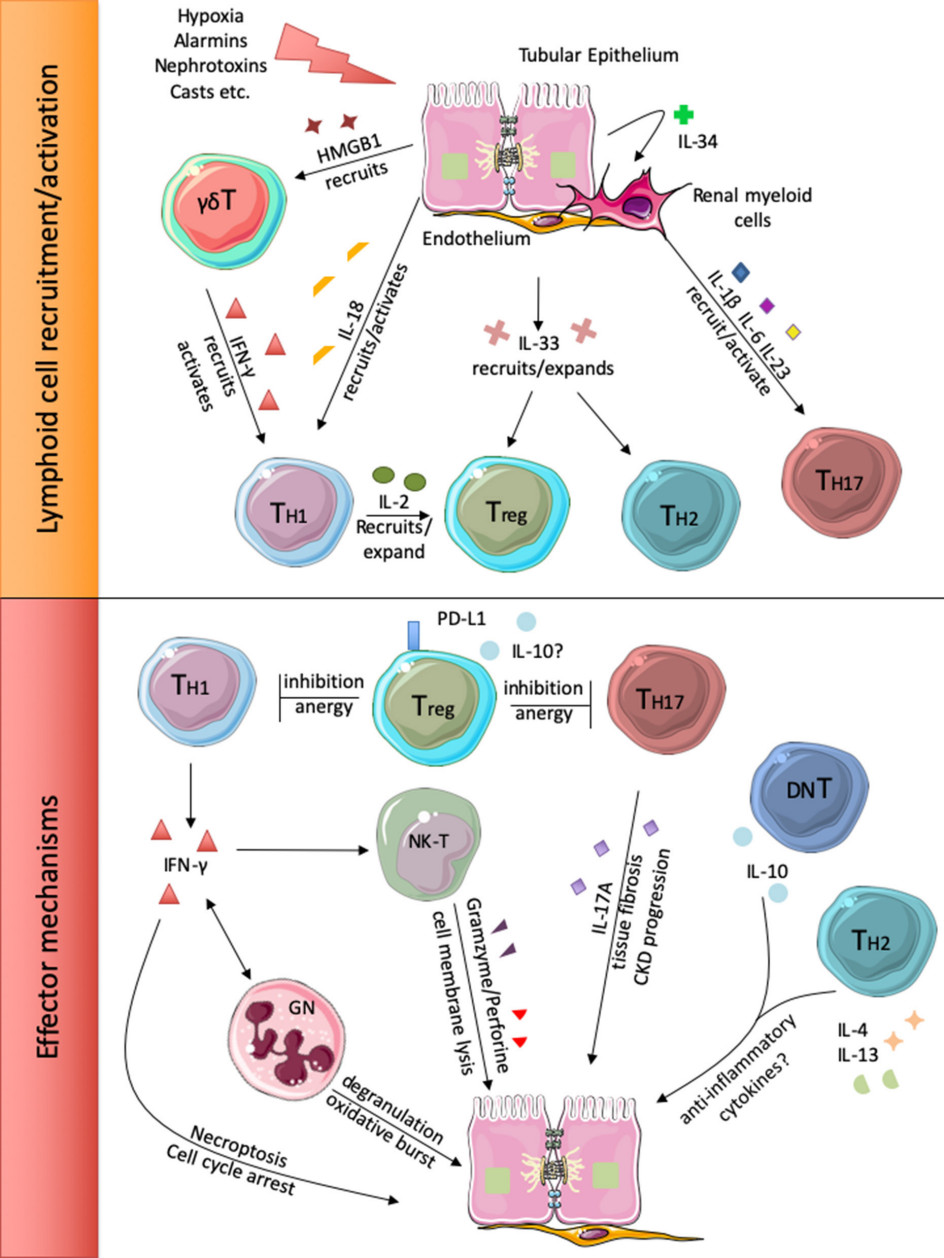
Injured renal cells release different alarm signals that recruit and activate local and circulating lymphoid cells (upper panel). Subsequently, the different lymphocyte subsets participate to renal injury perpetration or inhibition (lower panel).

AKI encompasses a broad spectrum of renal insults causing decreased filtration. In the last decade, multiple classifications were proposed to identify and study underlying conditions (2). From an epidemiological point of view, an important difference exists between community acquired vs. hospital related AKI. Community-AKI is more likely pre-renal and usually occurs in older or medically compromised patients from dehydration or from drugs that limit glomerular perfusion (e.g., non-steroidal anti-inflammatory drugs or inhibitors of the renin angiotensin aldosterone axis) (3). Conversely, hospital-acquired AKI is more often intrinsic and more likely to be severe. Another classification identifies major clinical syndromes and procedures that have a strong causative link with AKI (e.g., sepsis related-AKI, post-cardiac surgery AKI etc.); the definition of these clinical settings may guide clinicians in the diagnostic and therapeutic approach. From an etiologic point of view, these AKI types share a large part of the underlying mechanisms (2) (Figure 1).

Sepsis is a leading cause of in-hospital AKI accounting for 30–50% of cases (4). During sepsis, microbial and released host products act as alarm signals (or alarmins) targeting pattern recognition receptors (PRR) (12). Renal endothelium, tubular epithelial cells (TEC) and immune cells express PRR that sense a wide variety injury related molecular motifs. PRR activation produces pro-inflammatory phenotypes in renal cells which also activate programmed cell death pathways. Immune cells migrate to the site of alarmin release and contribute to local inflammation. In addition to infection consequences, patient courses are further complicated by nephrotoxic drugs (e.g., aminoglycosides) and sepsis-related ischemic injury, discussed in more depth below.

Ischemia-reperfusion injury (IRI) is probably the most studied experimental AKI model. Clinically, it occurs from severe renal hypoperfusion caused by blood loss, septic shock, and other anatomical abnormalities of renal blood supply. Some authors classify post-surgical AKI as a distinct entity due to predictable complicating factors of alarmin release (as consequence of bacterial contamination or tissue damage) or nephrotoxic drug administration (e.g., contrast dye) (13). In animal models, protracted IRI induces acute tubular necrosis (ATN), a lesion characterized by the extensive necrosis of the proximal tubular segments at the corticomedullary junction. However, renal biopsies from patients with ischemic AKI show mild parenchymal damage, despite severe organ dysfunction; thus, since 2000, more studies hypothesize microvascular failure and forms of cellular dysfunction (e.g., loss of polarity, epithelial leaking, organelle injury etc.) being predominant features in humans (14).

Multiple interacting etiologies contribute to cancer-related AKI. Oncologic patients suffer AKI from combinations of tumor lysis syndrome, infections, procedural complications, neoplastic renal invasion, paraproteinemia (mostly related to plasma-cell cancers) and drug toxicity. Traditionally, alkylating agents most frequently caused ATN (e.g., platin compounds). In the last decade new agents, most prominently immune checkpoint blockade inhibitors, are increasingly used. Multiple studies showed how immune checkpoint inhibitors can trigger intra-renal inflammation and autoimmune renal damage (15).

AKI frequently occurs in hospitalized patients but is rarely the cause for admission and frequently occurs from distant organ injury/dysfunction. In hepatorenal syndrome, for example, vasoactive aminic metabolites accumulate in liver failure and produce renal circulatory failure (8). Subtypes of acute cardiorenal syndromes involve renal injury resulting from decreased cardiac output or venous congestion, while others involve cytokine release and neurohormonal changes. Other relevant detrimental cross-talk are mediated by lung (hypoxia, cytokine release), brain (natriuretic peptides, cytokines), or intestine (leaking of bacteria and toxic metabolites) (12). Finally, massive muscle cell lysis seen in crush injuries cause injury. Cell lysis byproducts produce electrolyte unbalance, alarmin release, increased circulating waste products, and myoglobin precipitation in tubular lumen (cast nephropathy) that can combine with dehydration and profoundly injury kidneys (16).

## Role of the Innate Inflammatory Response in AKI Pathogenesis

Innate immune cells perpetrate AKI damage directly (e.g., neutrophil degranulation) or by recruiting the adaptive immune cells. At baseline, renal tissue hosts mainly macrophages and dendritic cells (DC), while immature monocytes and neutrophils migrate to the kidney in response to the alarm signal.

### Neutrophils

Neutrophils are the most abundant leucocytes infiltrating the kidney immediately after IRI (17) and multiple studies have shown that in-hospital risk of AKI positively correlates with the percentage of circulating neutrophils, suggesting an involvement of these cells in AKI pathogenesis (18). In response to stress, tubular cells acutely release IL-18 which recruits and activates neutrophils (19). Activated renal endothelial cells express E-selectins that bind neutrophil β-integrins and initiate the diapedesis process (20). Neutrophils damage renal tissue by degranulation, IFN-γ release and by recruiting of NK T-cells (21).

### Macrophages

Macrophages are the most abundant kidney resident immune cells. During AKI, TEC acutely release IL-34 that activates resident macrophages and promotes tubular injury (22). TEC damage induces also the recruitment and the activation of circulating monocytes (23), which perpetrate the injury by releasing pro-inflammatory cytokines as IL-1β, TNF-α, IL-6, and IL-12 (24). Subsequently, both resident and infiltrating macrophages switch to the regulatory phenotype and promote tissue repair. In particular, resident macrophages develop an embryonic-like gene signature soon after IRI and secrete WNT4, which in turn activates β-catenin pathway in TEC and promotes their proliferation (17, 23).

### Dendritic Cells and NK Cells

Resident renal dendritic cells (DC) are the predominant source of TNF-α during AKI and promote T_H1_ activation by secreting IL-12p40 (25, 26); moreover, they release T_H17_ inducing cytokines as IL-1 and IL-23 (27).

Natural killer (NK) cells migrate to the kidney in inflammatory conditions and induce TEC damage by releasing perforin (28). A subset of NK expresses an invariant form of T cell receptor (NKT or invariant NK) and substantially contributes to interferon production after renal IRI (21).

### Complement System

Complement cascade is acutely activated during AKI and contributes to renal damage, as indicated by data from C3 knock-out mice that are protected from IRI (29). Human and murine data have shown that complement get activated during AKI trough the alternative (30) and the mannose binding leptin pathways (31). Additionally, C3 can be activated in kidney parenchyma after binding ammonia (amidic-C3) (32). Complement activation injuries perpetrates renal injury trough the generation of membrane attack complex, the recruitment of immune cells and the activation of C3b and C5b receptors on tubular and endothelial cells (33).

## Adaptive Immune Response

Most studies investigating the role of T cells as AKI mediators focus on CD4 (i.e., T helper cells) while CD8 (cytotoxic) involvement is controversial (see below). Depending on inflammatory context, naïve CD4 cells differentiate to T-helper (T_H_) 1, 2, 17, or regulatory T cells (T_reg_). After AKI, TEC primarily release T_H1_ inducing signals and renal myeloid cells polarize toward T_H17_; T_H2_ activation during AKI is less understood.

## T_H1_ Cells

T_H1_ responses are orchestrated by master regulator transcription factor T-bet and characterized by expression of surface marker CXCR3. T_H1_ are classically associated with IFN-γ secretion and responses to intracellular pathogens. IFN-γ is a cytokine with a ubiquitous receptor promoting MHC expression, autophagy, reducing cell proliferation, and activating inflammatory death pathways (e.g., necroptosis and pyroptosis) (34). T_H1_ differentiation and activity are promoted by IL-12, IL-18, and IFN-γ itself. Upon injury, TEC release IL-18, leading to conversion of naïve CD4 T cells into T_H1_ (35). Rapid intrarenal migration of IFN-γ+ CD4 cells after LPS injection is prevented in IL-18 receptor deficient mice that, in turn, limits kidney injury (36). Cytokines alone cause cell injury, but T_H1_ cells also recruit other immune cells. Li et al. reported T_H1_ rapidly travel to ischemic kidneys and promote neutrophil and NK chemotaxis (that peaks after 3 h) (21). Additionally, IFN-γ alters TEC making them apro-inflammatory via expression of costimulatory molecules ICOS-L and B7-1 (37), preventing TEC proliferation (38), and promoting their death by necroptosis (39).

Although T cells promote inflammatory transformation of TEC, cytokine dependent damage appears predominantly based on T_H1_ responses. Day et al. observed that infiltrating lymphocytes were the main source of IFN-γ, while the cytokine fraction released by TEC was dispensable (40) in experimental models. Human data is corroboratory: in a multicenter prospective study on 1,400 patients undergoing cardiac surgery, post-operatory increase in serum IFN-γ significantly and directly associated with AKI incidence and 1-year mortality (41). Rather than non-specific responses to damage associated molecular patterns (DAMP) experimental evidence points to antigen-specific T cell function. Renal injury was worse after IRI in T-cell depleted mice reconstituted with heterogeneous CD4 cells compared to those given monoclonal ones (42).

## T_H2_ Cells

Little is known regarding T helper 2 (T_H2_) cells during AKI. T_H2_ constitutively express transcription factor GATA3 and surface marker Crth2 (CCR4 in mice): canonically, these cells orchestrate the anti-parasitic immune response via IL-4 and IL-13 secretion and are associated with asthma and allergic diseases. Yokota and colleagues induced IRI in mice lacking the transcription factors STAT4 or 6; the first is essential for T_H1_ response while STAT6 induces T_H2_ phenotype (43). While STAT4 deficient mice were protected from AKI, STAT6 knock down associated with worse outcomes. Increased tubular injury in IL-4 deficient mice further supports a renoprotective designation for T_H2_ responses (43). Conversely, human data from post-cardiac surgery patients showed direct relationships between IL-4 and IL-13 serum concentrations, AKI incidence, and 1-year mortality (41). Clearly, further experimental and clinical evidence are required to understand how T_H2_ responses affect AKI.

## T_H17_ Cells

T helper 17 cells (T_H17_) are a subset of CD4 lymphocytes characterized by expression of IL-17A, IL-17F, and the transcription factor ROR-γt. T_H17_ are frequently identified by surface marker CCR6, with variable expression of IL-23R, CCR4, and CCR2 (humans) or CCR7 (mice) (27). In both humans and mice, naïve CD4 experimental polarization is driven by TGF-β, IL-6, IL-21, and IL-23. Other stimuli contribute to mature T_H17_ activation: angiotensin II, salt excess and IL-1β (27, 44). T_H17_ cells are particularly abundant in barrier epithelia (e.g., skin, gut) and respond primarily to fungal and extracellular bacterial infections by recruiting neutrophils and activating epithelium via IL-17. T_H17_ are linked to various autoimmune diseases; the prototypical T_H17_ disease is psoriasis and it is effectively treated with anti-IL-17 therapies (45).

More recently, T_H17_ cells emerged as main players in AKI pathophysiology. Different groups demonstrated that T_H17_ are the most abundant kidney infiltrating lymphocytes infiltrating following AKI in mice (27, 44). Pindjakova et al. observed that resident dendritic cells and TEC release IL-1 (α and β), IL-23, and IL-6 to promote intrarenal IL-17 migration and activation after AKI from ureteral obstruction. IL-1 signaling dominates the phenomenon and its suppression pushes lymphocytes phenotypes to T_H1_ (27). Mehrota and colleagues demonstrated that T_H17_ cells expressing calcium channel Orai1 are solely responsible for IL-17 production after IRI and, ultimately, for renal injury (46). A 10-fold increase in circulating Orai1^+^ T_H17_ cells are found in ICU patients with AKI compared to those without (46). *In vivo*, intra-renal expression of Orai1 persisted for days after AKI resolution and its inhibition prevented the transition to chronic kidney disease (CKD) (46). Intriguingly, the kidney also possesses mechanisms to counteract T_H17_ cell activation. Our group observed that erythropoietin (EPO), a kidney produced hormone, prevents T_H17_ induction (47) and ameliorates renal injury in a murine model of Balkan nephropathy. Together with studies demonstrating EPO prevents IRI, current experimental evidence strongly support inhibition of dominant T_H17_ responses are feasible to prevent AKI and related progression to CKD.

## T Regulatory Cells

CD4 regulatory cells (Treg) are immunosuppressive T cells characterized phenotypically by constitutively high levels of IL-2 receptor (CD25) expression and maintained functionally via transcription factor FOXP3.

Research suggests that Treg attenuate AKI (48). Jaworska and colleagues observed IRI amelioration after Treg transfer, an effect that was dependent on programmed death ligand 1 and 2 (PD-L1/2) expression by Treg. The relevance of PDL to renal inflammation is supported by experimental demonstration of PD-1 expression by tubular cells (49) and clinical observations of renal adverse events in patients treated with immune checkpoint inhibitors targeting PD-1/PD-L1 axis (50).

After injury, renal Treg inhibit inflammation in multiple ways; they release TGFβ and IL-10, halt production of IL-1β, TNF-α, and IFN-γ, and reduce overall CD4 proliferation (51). It is unclear which between intra-renal or circulating Treg represent the active pool during AKI, though evidence exists supporting both central and peripheral sources. Investigators observed that DC heat shock protein 70 (HSP70) production increased splenic Tregs that migrated to the kidney and attenuated IRI (52), while another recent paper pointed out the role of renal resident Treg during IRI (53). The authors observed a progressive increase in CD3^+^ T cells after ischemia that paralleled the development of tissue fibrosis. Among the most expanded subsets there was a resident Treg population that was characterized by the expression of IL-33 receptor, a marker usually associated with T_H2_ phenotype. The administration of IL2 and IL33 at the time of IRI activated this population, promoted rapid recovery, and prevented tissue fibrosis. Of note, a previous study demonstrated that IL-33 is released by renal endothelium after cisplatin administration (54). Given these conflicting results, the answer may be model dependent.

## Other T Cells (Gamma-Delta, Double Negative, CD8)

Nomenclature of T cells is receptor based: αβ T cells constitute the majority (i.e., T_H1_, T_2_, T_H17_, and Treg subsets) while γδ T cells, resident in skin and the gut epithelia, constitute <1% of peripheral T cells. Their effector responses, based on still undefined antigens, include release of IL-17, IFN-γ, and TNF-α. In murine IRI, γδ lymphocytes rapidly infiltrate kidney tissue and promote subsequent migration of αβ cells; of note, γδ depletion delays but does not prevent injury, while αβ T cell ablation is protective (55). This suggests γδ T cells affect kinetics of kidney injury but are not necessary for AKI. In a clinical study of 20 patients undergoing abdominal aortic repair, magnitude of γδ T cells disappearance from the circulation was proportional to kidney injury markers (56). The same pattern was seen experimentally in mice where TEC HMGB1 release induced γδ T cell kidney migration, supporting the paradigm that early γδ T cell AKI responses facilitate αβ T cell recruitment.

CD8 T cells role in AKI is less defined, if it exists. A 2001 study found no significant pathogenic role for CD8 cells in IRI (57). A subsequent paper reported that CD8 deficient mice were mildly protected from cisplatin induced AKI, but less than those with CD4 depletion (58). Finally, in a study on acute aristolochic acid nephropathy, authors reported both CD4 and CD8 depletions were *detrimental*. In particular, absence of CD8 cells was associated to higher intra-renal TNF-α production and reduction of anti-inflammatory macrophages (59). More work is required to more clearly define how, and if, CD8 cells affect AKI.

Double negative (DN) T cells represent an early stage of T cell maturation lacking CD4 and CD8 expression. DN T cells are ubiquitous and some authors hypothesize they are an independent differentiated population rather than a maturation stage (60). These lymphocytes constitute more than 30% of kidney T cells at baseline and rapidly proliferate after tubular damage. Martina et al. reported that DN cells secrete IL-10 after IRI thus being anti-inflammatory (61) and, ostensibly, protective.

## B Cells

Experimental B-cells work has not definitively defined their role in AKI. One IRI study with B-cell deficient mice showed decreased AKI. Serum from control mice having restored AKI; these results suggests an antibody-mediated mechanism (62). Renner et al. observed the opposite effect; B-cell deficient mice had less intra-renal IL-10 production and a worse renal outcome (63). Of note, the same study reported a harmful B-cell subset; natural-IgM from peritoneal lymphocytes precipitated on the glomerular basal membrane and activated the complement alternative pathway. Conversely, Lobo and Okusa reported that infusion of natural-IgM was actually protective from IRI by inducing B-regulatory cells (64). These conflicting studies are emblematic of ongoing conflict regarding B cells and AKI.

Larger consensus has been reached about the B-cell role in post-AKI renal fibrosis (65). It has been shown that fibroblasts increase their collagen production in tissues with a higher B cell signature (66). Consistently, B-cell depleting therapy (anti-CD20) prevented kidney interstitial fibrosis after ureteral obstruction (67).

## Immune Therapies in AKI

The important effects adaptive immune responses have in AKI pathogenesis suggest, that immune modulatory therapies might effectively achieve clinically desirable results. Pechman and coworkers observed that mycophenolate mofetil (an immunosuppressive agent that inhibits purine synthesis in lymphocytes) prevented AKI long-term sequalae as renal fibrosis and salt-sensitive hypertension (68). Experimental models further link T cells, MMF, and CKD; a murine study modeling AKI transitions to CKD, showed an inverse association between mycophenolate treatment vs. T_H17_ proliferation and CKD (69). Clinically relevant approaches preventing T_H17_ responses (i.e., EPO receptor agonism and Orai1 inhibition) effectively halted kidney disease in murine models. Moreover, targeting T_H17_ effector molecule, IL-17, prevented calcineurin inhibitor related renal fibrosis (70). Taken together, these findings justify further studies targeting T_H17_ responses to prevent CKD.

A promising approach to treat AKI is the promotion of endogenous Treg expansion. A known strategy promoting Treg expansion involves IL-2 function. The IL-2/anti-IL2 complex is a mixture of IL-2 with an antibody that prevents IL-2 binding to the β-chain of its receptor (CD25); this complex strongly induced Treg proliferation and attenuated IRI by reducing neutrophil and macrophage migration in renal tissue (71). Other authors generated a fusion IL-2/IL-33 cytokine that expanded intra-renal IL-33R^+^ Treg, halted CD4 effector cell proliferation and prevented 100% of the observed mortality in a murine IRI model (72). Dimethylsphingosine (DMS) promotes CD4 migration to kidneys at baseline and after ischemia; Lai et al. demonstrated that FOXP3+ lymphocytes were proportionally more abundant in the renal tissue after DMS treatment and prevented IRI in mice (73).

Treg adoptive transfer effectively downregulates IRI and other types of renal injury in pre-clinical models (48); however, its clinical application remains challenging. Therapeutic cell products need specific cell factories, are temperature and time-sensitive (thus needing complicated stock and transport procedures), are prone to contamination, may degenerate in neoplastic disease and can trigger the host immune response toward allo-antigens (73).

Stem cell therapies have been proposed for the management of AKI; in particular, mesenchymal stromal cells (MSC) have been successfully used in different preclinical models and are currently under investigation in clinical trials (74). MSC are immune-regulatory; importantly, MSC infusion expands intra-renal Treg after IRI. Consequent reductions in circulating IL-6, TNF-α and IFN-γ levels are Treg dependent (75). Treg strategies, therefore, intersect with other established experimental protocols.

However, cell infusion poses previously mentioned challenges, even if MSC are relatively easy to expand and not immunogenic. An interesting alternative comes from the observation that MSC conditioned medium is as effective as MSC infusion in promoting tissue regeneration (76). Indeed, MSC beneficial effects in AKI are not contact-mediated and MSC do not differentiate in any mature kidney cell. If future efforts identify substances inducing intra-renal Treg expansion therapies would avoid cell infusion complications.

## Conclusions

Inflammation produces AKI via reciprocal interactions between renal parenchyma, resident immune cells, and recruited immune cells. Increasing recent evidence indicates a dominant role of the adaptive immune response, and T cells in particular, as prominent pathogenic elements as well as mitigating factors. Myriad AKI etiologies frequently condense into recurrent identifiable immune patterns associated with tubular injury and T cells. In particular, CD4 and γδ T cells are initial immune effectors migrating to kidneys and orchestrating activation of innate cells. Early injury phases are characterized by a strong IFN-γ response, possibly produced by T_H1_ cells. In later phases, T_H17_ perpetuate injury and tissue fibrosis. Conversely, Treg and possibly T_H2_ exert opposing anti-inflammatory roles and limit or prevent injury. Pre-clinical and observational studies provide strong bases of feasibility for future pharmacological interventions targeting lymphocyte function to prevent and limit AKI as well as subsequent renal fibrosis.

## Author Contributions

SD wrote the first draft. JL reviewed it. PC had the original idea and reviewed the manuscript. All authors approved the final manuscript.

## Conflict of Interest

The authors declare that the research was conducted in the absence of any commercial or financial relationships that could be construed as a potential conflict of interest.
